# Self-Referred Late-Onset Hypogonadism Hypothesis and Testosterone Replacement Therapy in Major Depressive Disorder Under Polypharmacy: A Case Report

**DOI:** 10.7759/cureus.103956

**Published:** 2026-02-20

**Authors:** Kenta Ichino, Nobuo Okui, Hisamitsu Ide, Shigeo Horie

**Affiliations:** 1 Data Science and Informatics for Genetic Disorders, Graduate School of Medicine, Juntendo University, Tokyo, JPN; 2 Urology, Graduate School of Medicine, Juntendo University, Tokyo, JPN; 3 Innovative Longevity, Graduate School of Medicine, Juntendo University, Tokyo, JPN; 4 Urology, Yokosuka Urogynecology and Urology Clinic, Kanagawa, JPN; 5 Mathematics, Kanagawa Dental University, Kanagawa, JPN

**Keywords:** diagnostic reasoning, diferential diagnosis, late-onset hypogonadism, major depressive disorder diagnosis and treatment, major depressive disorder (mdd), psychiatric polypharmacy, psychiatry sexual dysfunction, self-reporting bias, symptom assessment, testosterone replacement therapy (trt)

## Abstract

Major depressive disorder (MDD) under treatment may be accompanied by residual sexual dysfunction and fatigue. This broadens the differential diagnosis and destabilizes symptom attribution. A 54-year-old Japanese man with MDD formed a late-onset hypogonadism (LOH) hypothesis from online information and self-referred to urology. He was receiving long-term polypharmacy and presented with fatigue, insomnia, arthralgia, and reduced libido. Total testosterone was 2.82 ng/mL, and free testosterone was 5.3 pg/mL. After counseling and informed consent, testosterone replacement therapy (TRT) was initiated with testosterone enanthate 250 mg intramuscularly once monthly. During eight injections, perceived benefit was limited and transient. Erectile difficulty and irritability recurred, and TRT was discontinued. Depressive symptoms persisted, and ongoing medication adjustments were required during TRT. This limited causal interpretation of symptom changes and TRT responsiveness. This case shows that reducing diagnostic reasoning to a binary choice of LOH or MDD can fragment hypothesis updating. From the outset, depressive pathophysiology, medication effects, and the LOH hypothesis should be evaluated in parallel. A shared diagnostic plan with prespecified indicators and discontinuation criteria can preserve diagnostic convergence even under fragmented care.

## Introduction

Major depressive disorder (MDD) is clinically heterogeneous, and symptom patterns vary even within the Diagnostic and Statistical Manual of Mental Disorders, Fifth Edition definitions [1,2]. In routine practice, MDD severity assessment often relies on summed scores from standardized symptom rating scales. This approach assumes that different symptom combinations indicate equivalent severity, and prior studies discuss that it can obscure clinically meaningful between-patient differences [2,3]. As treatment progresses, symptom heterogeneity becomes more apparent and can complicate the attribution of residual symptoms and clinical decision-making [4].

In men presenting with depressive symptoms, sexual dysfunction such as decreased libido and erectile dysfunction may become clinically prominent [5]. These symptoms are closely associated with quality of life (QOL) and can influence both symptom persistence and healthcare-seeking patterns [5,6]. In particular, residual symptoms such as sexual dysfunction and fatigue after therapeutic intervention can be attributed to the persistence of the primary disorder, coexisting pathology, or treatment-related factors. This structure readily promotes expansion of the differential diagnosis.

Late-onset hypogonadism (LOH) is conceptualized as a clinical and biochemical syndrome associated with low testosterone [7-10]. Highly specific symptoms include decreased libido and erectile dysfunction. LOH can also include nonspecific symptoms such as fatigue, cognitive complaints, and depressed mood [5,9]. Therefore, when symptoms such as sexual dysfunction and fatigue persist during MDD treatment in men, it is clinically reasonable to consider LOH in the differential diagnosis. However, symptom overlap creates a situation in which symptoms alone do not yield diagnostic convergence. This can generate instability in diagnostic attribution. Clinical diagnosis of LOH requires compatible symptoms and consistently low testosterone. Guidelines recommend measuring morning total testosterone (TT), ideally under fasting conditions, and repeating testing to confirm low values. Additional evaluation may include measuring gonadotropins and sex hormone-binding globulin, and assessing free testosterone (FT) when indicated, to interpret testosterone levels and to distinguish primary from secondary hypogonadism [9,10].

Within the treatment context, pharmacotherapy can further complicate symptom attribution. Antidepressants and other psychotropic agents are associated with sexual dysfunction and changes in sleep, energy, and affect, which overlap with core symptom domains of MDD and LOH [11-13]. In routine practice, patients may also receive concomitant non-psychiatric medications, some of which have debated or uncertain relationships with sexual function. Consequently, persistent symptoms such as sexual dysfunction and fatigue during ongoing MDD treatment can plausibly be attributed to illness persistence, comorbidity, or medication effects. This interpretive flexibility can expand the differential diagnosis and destabilize diagnostic attribution.

In recent years, international interest in LOH and testosterone therapy has increased [14,15]. Meanwhile, the clear indication for testosterone therapy is replacement therapy for hypogonadism, and its application to men without pathological hypogonadism remains a contested area [16]. This social interest and the surrounding information environment can influence patient self-interpretation and healthcare-seeking behavior, including specialty selection.

This case report examines how symptom overlap, medication exposure, and patient-initiated specialty access destabilize diagnostic attribution between MDD-related symptoms, medication effects, and an LOH hypothesis.

## Case presentation

Patient information

The patient was a 54-year-old Japanese man (aged 49 at the first psychiatric visit). His height was 168.4 cm, and his weight was 48 kg at both the initial psychiatric and initial urological visits. His body mass index was 16.9 kg/m². Self-reported premorbid personality characteristics included a pragmatic and meticulous style, marked worry-proneness and perfectionism, a strong sense of responsibility, a taciturn manner, and difficulty engaging in interpersonal interactions. He reported no alcohol use and was a former smoker who quit 20 years earlier. He was unmarried and lived with both parents, serving as the primary caregiver for both parents. His father died in 2022 due to hepatic encephalopathy, and caregiving for the currently bedridden mother continued. He resigned from employment in 2012 to provide caregiving duties for his parents, followed by unemployment, supported by public assistance. After the first presentation to the hospital in 2020, multiple chronic conditions were diagnosed and treated across several departments, and each remained stable under ongoing management (Table 1).

**Table 1:** Major timeline of the case. At each time point, psychiatric symptoms and functional status, major clinical events, interventions, and selected test findings are summarized to document longitudinal changes and management. Detailed blood test results are provided in Table 2. Only major events and clinically relevant tests are shown. A dash (—) indicates none. HAM-D: Hamilton Depression Rating Scale; SI: suicidal ideation; LOH: late-onset hypogonadism; Hb: hemoglobin; Hct: hematocrit; PSA: prostate-specific antigen; LH: luteinizing hormone; FSH: follicle-stimulating hormone; PRL: prolactin; TT: total testosterone; FT: free testosterone; TRT: testosterone replacement therapy; GERD: gastroesophageal reflux disease

Time point	Psychiatric symptoms/function	Major events	Interventions	Tests
2012/4/1	Depressive symptoms onset reported by the patient	Resigned for family caregiving; public assistance	—	—
2020/2/1	—	Dermatology—androgenetic alopecia (AGA)	Finasteride 1 mg/minoxidil initiated	—
2020/5/1	—	Orthopedics—tenosynovitis	—	—
2020/7/1	HAM-D score 7 (Depressed mood — 2 points; Suicide — 2 points; Insomnia, early — 1 point; Work and activities — 1 point; Somatic anxiety — 1 point)	First psychiatry visit; unemployment/caregiving burden; difficulty adapting to environment	Quetiapine 25 mg initiated	ECG/blood tests/non-contrast head CT: no abnormalities
2020/12–2021/02	Insomnia/anxiety improved (partial); depressive symptoms persisted; SI persisted	Pulmonology—asthma; Otolaryngology—allergic rhinitis	2020/12 Sertraline 25 mg/ICS/LABA initiated; 2021/01 sertraline 50 mg increased; 2021/02 alprazolam/suvorexant initiated	—
2021/04–2021/06	Affective lability/SI worsened	Increased caregiving burden	Quetiapine 100 mg increased	—
2021/7/1	Fatigue; insomnia; arthralgia; reduced libido; erectile function preserved	First urology visit; concern about LOH	—	Hb 14.8 g/dL; Hct 44.2%; PSA 0.763 ng/mL; LH 3.1 mIU/mL; FSH 5.3 mIU/mL; PRL 6.8 ng/mL; TT 2.82 ng/mL; FT 5.3 pg/mL (12:00)
2021/08–2022/04	Depressive symptoms/affective lability persisted; perceived mild improvement in some months during early TRT; overall, no perceived benefit; difficulty maintaining erections	Caregiving burden increased; father’s condition worsened, and frequent admissions/discharges	2021/08 Quetiapine 200 mg/sertraline 100 mg/alprazolam 1.6 mg increased; 2021/10 quetiapine 250 mg/alprazolam 2.4 mg increased; 2022/02 hochuekkito initiated; 2021/08-2022/03 TRT	—
2022/07–2022/11	Follow-up/medication interruption; depressive symptoms/insomnia persisted; appetite decreased; weight loss trend	2022/07 Father died (hepatic encephalopathy); 2022/08 medications restarted; 2021/12 dermatology follow-up interrupted; 2021/12 Gastroenterology—GERD	2022/08 Sertraline 50 mg reduced; alprazolam discontinued; 2022/11 sertraline discontinued; mirtazapine 30 mg/lorazepam 0.5 mg initiated; suvorexant discontinued	—
2023/02–2023/06	Appetite decreased; insomnia persisted; depressive symptoms/affective lability worsened	2023/04 Mother hospitalized (cerebral infarction); transported by syncope; 2023/06 Mother discharged; bedridden	2023/02 Quetiapine discontinued; olanzapine 10 mg initiated; mirtazapine 45 mg increased; 2023/04 olanzapine 12.5 mg increased; 2023/06 lemborexant 5 mg initiated; mirtazapine 30 mg reduced	ECG/blood tests/non-contrast head CT (outside institution): patient-reported “no abnormalities,” records unavailable
2023/12–	2023/12 agitation/anxiety/pessimistic remarks; 2024/04 SI worsened; risperidone effective	2023/12 Orthopedics—cervical/lumbar spondylosis; 2024/06 Dermatology—AGA (treatment restarted)	2023/12 Olanzapine 20 mg increased; loxoprofen sodium/trigger-point injection initiated; 2024/04 risperidone 1 mg as needed initiated; 2024/06 finasteride 1 mg restarted	2024/02 TT 3.87 ng/mL (12:00); 2025/04 TT 5.61 ng/mL (12:00)
Current	—	—	Mirtazapine 30 mg; olanzapine 20 mg; lorazepam 0.5 mg; lemborexant 5 mg; risperidone 1 mg as needed; hochuekkito; finasteride 1 mg	—

Psychiatric course

Depressive symptoms began around 2012 by self-report, and he regarded prolonged unemployment as the primary contributing factor. In July 2020, he presented to the psychiatry department with depressed mood as the chief complaint. At the initial psychiatric evaluation, he was polite and calm with appropriate eye contact, but was talkative with psychomotor agitation. Appetite was maintained. Sleep was described as light but lasted approximately six hours. The Hamilton Depression Rating Scale score (HAM-D) was 7, and suicidal ideation (SI) was present. Vital signs were a temperature of 36.7°C, blood pressure of 112/86 mmHg, heart rate of 78 beats/minute, and respiratory rate of 18 breaths/minute, with no abnormal neurological findings on examination. No evidence of mania was identified. Blood tests showed no clinically significant abnormalities (Table 2), and non-contrast head CT revealed no significant findings. A diagnosis of MDD was made based on the reported history, symptom profile, including depressed mood, markedly diminished interest or pleasure, sleep disturbance, reduced activity level, feelings of worthlessness, impaired concentration, SI, and functional impairment.

**Table 2:** Longitudinal blood test results. Serial blood test results during follow-up. Values are reported by test date. Collection times are shown when recorded. A dash (—) indicates not measured. WBC: white blood cell count; RBC: red blood cell count; Hb: hemoglobin; Hct: hematocrit; PLT: platelet count; ALP: alkaline phosphatase; AST: aspartate aminotransferase; ALT: alanine aminotransferase; LDH: lactate dehydrogenase; γGTP: gamma-glutamyl transpeptidase; CK: creatine kinase; tBil: total bilirubin; dBil: direct bilirubin; TP: total protein; Alb: albumin; BUN: blood urea nitrogen; Cr: creatinine; eGFR: estimated glomerular filtration rate; UA: uric acid; TG: triglycerides; TChol: total cholesterol; HDL: high-density lipoprotein cholesterol; LDL: low-density lipoprotein cholesterol; Glu: glucose; Amy: amylase; Lipa: lipase; Na: sodium; K: potassium; Cl: chloride; Ca: calcium; P: phosphate; Zn: zinc; CRP: C-reactive protein; HbA1c: glycated hemoglobin; TSH: thyroid-stimulating hormone; fT3: free triiodothyronine; fT4: free thyroxine; PSA: prostate-specific antigen; LH: luteinizing hormone; FSH: follicle-stimulating hormone; PRL: prolactin; CORT: cortisol; TT: total testosterone; FT: free testosterone

Test	Unit	2020					2021		2024	2025
		1/16	3/25	7/9	7/13	9/23	3/15	7/5	2/19	4/28
		—	—	—	—	—	—	12:39	11:48	12:38
WBC	/µL	7,300	5,000	5,200	6,100	4,800	5,400	6,100	5,500	6,100
RBC	/µL	4.57	4.6	4.58	4.59	4.37	4.3	4.8	4.84	4.46
Hb	g/dL	14.2	14.5	14.8	14.6	13.7	13.1	14.8	15.1	14.4
Hct	%	43	42.5	43	43.3	41.7	40.1	44.2	46.6	43.8
PLT	/µL	25.2	20.3	19.6	21.6	16.8	31.2	23.8	32.8	25
ALP	IU/L	—	—	—	—	—	—	114	92	86
AST	IU/L	20	22	23	18	24	20	15	20	16
ALT	IU/L	14	21	22	18	35	24	14	20	18
LDH	IU/L	179	184	172	—	178	223	166	196	200
γGTP	IU/L	8	9	8	—	8	9	9	11	10
CK	IU/L	—	—	—	62	—	—	—	—	—
tBil	mg/dL	—	—	—	0.88	—	0.63	0.9	1.07	0.53
dBil	mg/dL	—	—	—	<0.05	—	—	—	—	—
TP	g/dL	7.1	6.9	7.2	7.3	6.9	6.7	7.3	7.2	7
Alb	g/dL	—	—	—	4.2	—	—	4.2	4	3.8
BUN	mg/dL	13	15	15	16	15	12	12	15	20
Cr	[mg/dL]	0.85	0.84	0.88	0.88	0.8	0.82	0.9	0.86	0.77
eGFR	mL/minute/1.73m^2^	76.3	76.8	73	73	81	78.4	70.8	73.2	82.2
UA	mg/dL	—	4.5	4.8	4.6	4.2	—	5.1	4.1	3.5
TG	mg/dL	—	—	—	—	—	—	46	—	—
TChol	mg/dL	—	—	—	119	—	—	140	—	—
HDL	mg/dL	—	—	—	44	—	—	46	—	—
LDL	mg/dL	—	—	—	—	—	—	75	—	—
Glu	mg/dL	94	—	—	—	—	98	169	109	—
Amy	IU/L	—	—	—	—	—	—	77	—	—
Lipa	IU/L	—	—	—	—	—	—	16	—	—
Na	mmol/L	140	140	143	142	141	—	141	142	142
K	mmol/L	3.8	4.3	3.6	3.9	3.8	—	3.9	4.3	4.4
Cl	mmol/L	103	105	105	103	106	—	101	106	106
Ca	mg/dL	—	—	—	9.1	—	—	—	9.7	9.2
P	mg/dL	—	—	—	3.6	—	—	—	—	—
Zn	µg/dL	—	—	—	—	—	—	64	—	—
CRP	mg/dL	—	—	—	<0.02	—	—	<0.02	—	—
HbA1c	%	—	—	—	5.2	—	—	5.4	5.5	5.5
TSH	µIU/mL	—	—	—	1.84	—	—	—	—	—
fT3	pg/mL	—	—	—	2.9	—	—	—	—	—
fT4	ng/dL	—	—	—	1.2	—	—	—	—	—
PSA	ng/mL	—	—	—	—	—	—	0.763	0.977	0.487
LH	mIU/mL	—	—	—	—	—	—	3.1	4.6	9
FSH	mIU/mL	—	—	—	—	—	—	5.3	5.4	7.6
PRL	ng/mL	—	—	—	—	—	—	6.8	—	—
CORT	nmol/L	—	—	—	—	—	—	7	—	—
TT	ng/mL	—	—	—	—	—	—	2.82	3.87	5.61
FT	pg/mL	—	—	—	—	—	—	5.3	6.6	11.1

After pharmacotherapy was initiated, sleep disturbance and anxiety showed partial improvement. However, depressed mood and SI persisted, and increasing caregiving burden was associated with affective lability and worsening sleep disturbance. Table 1 summarizes the chronological psychiatric course, including psychiatric symptoms/function, major events, interventions, and tests. In April 2023, after the mother was hospitalized for a cerebral infarction, he experienced syncope and was transported to the emergency department. According to his report, ECG, blood tests, and a non-contrast head CT were performed at an outside institution, and he was informed that no clinically significant abnormalities were identified. Following this episode, his MDD symptoms worsened and required further medication adjustment. From 2024 onward, outpatient follow-up continued with fixed medication doses. SI persisted from the initial visit, but there was no history of suicide attempts and no concrete plan. Caregiving responsibility functioned as a protective factor, and he understood this role and was able to commit to not acting on SI.

Urological course

In July 2021, he visited the department of urology after reading about male andropause-related symptoms and thinking his symptoms were consistent with LOH. Presenting complaints included fatigue, insomnia, arthralgia, and reduced libido, while erectile ability was preserved. Laboratory evaluation included a TT of 2.82 ng/mL and an FT of 5.3 pg/mL, with blood sampling performed at 12:00. Detailed values are summarized in Table 1. The patient was the primary caregiver for his parents, and clinic attendance was restricted to midday hours. Early-morning fasting sampling was not possible under these constraints. Repeat confirmatory early-morning fasting TT testing was not obtained before initiating testosterone replacement therapy (TRT), which reduced biochemical reproducibility and diagnostic certainty. Given ongoing MDD treatment and concurrent medications that can affect sexual function, LOH remained one of several competing hypotheses for reduced libido and fatigue. After counseling and obtaining informed consent, a trial of TRT was initiated.

TRT consisted of testosterone enanthate 250 mg administered intramuscularly once monthly, administered eight times from August 2021 through March 2022, and discontinued in April 2022. Efficacy assessment relied on patient-reported changes in mood, activity, and sexual symptoms. Standardized depression or sexual-function scales were not collected during TRT. Self-reported status at each visit and the clinical course are summarized in Table 3. Psychotropic medication doses were also adjusted during the TRT period (Table 1), which complicated attribution of symptom changes to TRT versus other factors. TT increased on subsequent time-matched measurements to 3.87 ng/mL (February 2024) and 5.61 ng/mL (April 2025). These follow-up TT measurements were also obtained at 12:00. They do not constitute guideline-recommended confirmatory morning testing. At the last urology follow-up, he continued hochuekkito, and he did not report worsening sexual dysfunction. Depressive symptoms remained stable. If sexual dysfunction emerges in the future, potential medication-related factors should be reassessed, and dose reduction can be considered.

**Table 3:** TRT course with patient-reported outcomes Monthly TRT course (August 2021–April 2022) with patient-reported effects, management decisions, and available TT measurements. A dash (—) indicates not measured. TRT: testosterone replacement therapy; TT: total testosterone

Time point	Session	Subjective effects	Concomitant therapy/plan	Tests
2021/8/1	1	Desired treatment	TRT initiated	TT 2.82 ng/mL
2021/9/1	2	Slight mood improvement; no marked perceived effect	Continued	—
2021/10/1	3	Slight mood improvement; no marked perceived effect	Continued	—
2021/11/1	4	No increase in motivation; difficulty maintaining an erection	Desired to continue	—
2021/12/1	5	Increased activity; difficulty maintaining an erection	Continued	—
2022/1/1	6	Increased activity; difficulty maintaining an erection	Continued	—
2022/2/1	7	Lacks energy	Desired to continue; hochuekkito initiated	—
2022/3/1	8	Irritability present	Desired to continue	—
2022/4/1	Discontinued	Irritability present; no perceived benefit	TRT discontinued	—

## Discussion

This case describes a patient receiving ongoing treatment for MDD with SI under polypharmacy. He formed an LOH hypothesis from media information and self-referred to urology without an internal referral, leading to initiation of TRT. At the initial urology visit, the chief complaints were fatigue, insomnia, arthralgia, and reduced libido. He initially perceived some benefit during TRT, but later reported recurrent erectile difficulty and irritability with diminishing perceived benefit, and TRT was discontinued. The key diagnostic issue is not the reduction of this course to a binary differential of LOH versus MDD. Instead, the case visualizes how a pre-existing psychiatric diagnosis, a patient-driven access pathway, medication-related factors, and a fragmented care process disperse hypothesis updating and interpretation of interventions.

LOH is generally diagnosed when compatible symptoms are accompanied by consistently low testosterone levels [10]. In contrast, symptoms suggestive of androgen deficiency, including sexual symptoms, are nonspecific, and comorbidities and other factors often contribute substantially to the presenting symptoms [5,9]. Diagnostic difficulty is further complicated by the frequent discordance between symptoms and low testosterone within the same individual [7]. In this case, psychiatric treatment preceded urological evaluation, and psychotropic medications such as sertraline, quetiapine or olanzapine, and mirtazapine were used long-term. Finasteride was also initiated by dermatology [17,18]. Therefore, when assessing sexual dysfunction and reduced libido, medication effects should be treated as a competing hypothesis. The evaluation plan should be structured into three parallel branches that include depressive pathophysiology, medication effects, and the LOH hypothesis.

A central feature of this case is that the patient interpreted his symptoms as consistent with online articles and therefore sought urological care by self-referral. Rather than treating the patient’s hypothesis formation as mistaken self-diagnosis, the online information environment should be incorporated as a contemporary determinant of care-seeking pathways and diagnostic framing. Recent discussions describe how the accumulation of online information contributes to a global surge in testosterone prescribing [16]. Studies also describe a substantial increase in testosterone prescribing among men without documented pathological hypogonadism [15]. Therefore, patient presentation with an LOH hypothesis is not exceptional, and the explanation and evaluation design should be reconstructed on that premise. Concretely, before anchoring on LOH as the explanatory label, it is useful to co-document a shared diagnostic plan [19]. This plan specifies what would strengthen or weaken the LOH hypothesis, what would support medication-related causality, and how factors that prolong depressive symptoms will be managed. This approach provides an anchor for diagnostic convergence even when access pathways branch under patient initiative.

This case involved patient-initiated visits across several specialties, including dermatology, orthopedics, pulmonary medicine, otolaryngology, urology, and gastroenterology. When consultations occur through patient self-referral without internal referral, information sharing is difficult to formalize, and responsibility for hypothesis updating becomes dispersed [19]. Coexisting MDD, medication effects, and an LOH hypothesis complicate attribution of symptom changes to specific interventions in this case. In practice, once TRT is initiated, psychiatric symptoms, sleep, daily routine, and concomitant medication changes can shift in parallel, further obscuring effect attribution. A practical implementation proposal is the introduction of a shared diagnostic plan. By sharing the competing hypotheses, the next indicators to assess, the priority order of interventions, and the criteria for discontinuation or modification, specialists can preserve the ability to distinguish intervention effects even in fragmented clinical management.

Before TRT initiation, a structured discussion of anticipated benefits, potential risks, and the monitoring plan is recommended [10]. TRT requires careful review of contraindications and cautions with appropriate monitoring. TRT is not recommended when near-term fertility is desired, and treatment selection therefore involves decision constraints [10]. Accordingly, even when TRT is used as a short diagnostic trial, the assessment should be prespecified at initiation by defining the target effect domains and the criteria for continuation, discontinuation, or modification. In this case, response assessment relied on patient-reported symptoms without standardized measures, limiting interpretability.

In this case, early improvement in mood was recorded during initial TRT, but a marked response did not occur, perceived benefit gradually diminished, and TRT was discontinued. Evidence for TRT effects on depressive symptoms is heterogeneous, with a systematic review of LOH suggesting benefit in mild depression but not in MDD [20]. Another review stated that the association between low testosterone and depression, as well as the antidepressant effect of TRT, remains inconclusive. Therefore, limited perceived benefit from TRT during MDD treatment should not be used to infer the absence of LOH. The key diagnostic contribution of this case is an algorithm for hypothesis updating when the response is insufficient. Specifically, it preserves the three-branch structure of depressive pathophysiology, medication effects, and the LOH hypothesis, and updates hypotheses in the following order. The sequence is (i) safety confirmation, including adverse events and monitoring items; (ii) medication relationships, including temporal relationships and modifiability of antidepressants, antipsychotics, and finasteride; (iii) factors that prolong MDD, including caregiving burden, SI, and functional impairment; (iv) sleep and circadian routine, including persistent insomnia and daytime activity; and (v) LOH re-evaluation using a symptoms-and-biochemistry framework, with standardized sampling conditions and repeat testing [9,10]. When discontinuation of causal medications is difficult, the plan should explicitly prioritize continuation of MDD treatment while managing symptomatic treatment and evaluation of the sexual-function domain on a separate track to prevent cross-domain information mixing.

There are several limitations. Clinic attendance was restricted to midday hours due to caregiving responsibilities, limiting standardized early-morning fasting sampling. Repeat confirmatory early-morning fasting TT testing was not obtained before TRT initiation, which reduced biochemical reproducibility and diagnostic certainty [9,10]. Assessment of TRT effects relied primarily on subjective reports rather than quantitative evaluations using standardized scales. Details of interdepartmental information sharing and coordination processes were unclear. Responsibility for hypothesis updating was prone to becoming dispersed.

## Conclusions

This case shows that single-hypothesis framing during MDD management with an LOH hypothesis can fragment hypothesis updating and obscures interpretation of interventions. A parallel framework that keeps depressive pathophysiology, medication effects, and the LOH hypothesis in view from the outset improves interpretability. In practice, a shared diagnostic plan can document findings that would support or weaken each hypothesis, the intervention order, and stopping rules. TRT is better described as a time-limited therapeutic intervention with prespecified evaluation domains and safety monitoring, rather than as a “diagnostic trial.” In this case, perceived benefit during TRT was insufficient to support exclusion of LOH, particularly given the limitations in biochemical confirmation. Re-evaluation may proceed by revisiting competing hypotheses, including medication-related factors and standardized endocrine reassessment when feasible. This approach preserves diagnostic convergence and interpretation of treatment response, even when care is fragmented.
